# Nutritional and Lifestyle Features in a Mediterranean Cohort: An Epidemiological Instrument for Categorizing Metabotypes Based on a Computational Algorithm

**DOI:** 10.3390/medicina60040610

**Published:** 2024-04-08

**Authors:** Aquilino García-Perea, Edwin Fernández-Cruz, Victor de la O-Pascual, Eduardo Gonzalez-Zorzano, María J. Moreno-Aliaga, Josep A. Tur, J. Alfredo Martinez

**Affiliations:** 1 General Council of Pharmaceutical Associations, 28001 Madrid, Spain; 2 IMDEA-Food Institute (Madrid Institute for Advances Studies), 28049 Madrid, Spain; 3 Faculty of Health Sciences, International University of La Rioja (UNIR), 26006 Logroño, Spain; 4 Consumer Healthcare Scientific Department, Cinfa Laboratories, 31620 Huarte, Spain; 5 CIBEROBN (Pathophysiology of Obesity and Nutrition), Carlos III Health Institute, 28029 Madrid, Spain; 6 Center for Nutrition Research and Department of Nutrition, Food Sciences and Physiology, University of Navarra, 31008 Pamplona, Spain; 7 IdISNA, Navarra Institute for Health Research, 31008 Pamplona, Spain; 8 Research Group on Community Nutrition and Oxidative Stress, University of the Balearic Islands-IUNICS, 07122 Palma de Mallorca, Spain; 9 IDISBA, Health Research Institute of the Balearic Islands, 07120 Palma de Mallorca, Spain; 10 Department of Medicine, Dermatology, and Toxicology, University of Valladolid, 47005 Valladolid, Spain

**Keywords:** metabotype, lifestyle assessment, healthcare, precision nutrition, cluster, machine learning

## Abstract

*Background and Objectives:* Modern classification and categorization of individuals’ health requires personalized variables such as nutrition, physical activity, lifestyle, and medical data through advanced analysis and clustering methods involving machine learning tools. The objective of this project was to categorize Mediterranean dwellers’ health factors and design metabotypes to provide personalized well-being in order to develop professional implementation tools in addition to characterizing nutritional and lifestyle features in such populations. *Materials and Methods:* A two-phase observational study was conducted by the Pharmacists Council to identify Spanish nutritional and lifestyle characteristics. Adults over 18 years of age completed questionnaires on general lifestyle habits, dietary patterns (FFQ, MEDAS-17 p), physical activity (IPAQ), quality of life (SF-12), and validated well-being indices (LS7, MEDLIFE, HHS, MHL). Subsequently, exploratory factor, clustering, and random forest analysis methods were conducted to objectively define the metabotypes considering population determinants. *Results:* A total of 46.4% of the sample (*n* = 5496) had moderate-to-high adherence to the Mediterranean diet (>8 points), while 71% of the participants declared that they had moderate physical activity. Almost half of the volunteers had a good self-perception of health (49.9%). Regarding lifestyle index, population LS7 showed a fair cardiovascular health status (7.9 ± 1.7), as well as moderate quality of life by MEDLIFE (9.3 ± 2.6) and MHL scores (2.4 ± 0.8). In addition, five metabotype models were developed based on 26 variables: Westernized Millennial (28.6%), healthy (25.1%), active Mediterranean (16.5%), dysmetabolic/pre-morbid (11.5%), and metabolically vulnerable/pro-morbid (18.3%). *Conclusions:* The support of tools related to precision nutrition and lifestyle integrates well-being characteristics and contributes to reducing the impact of unhealthy lifestyle habits with practical implications for primary care. Combining lifestyle, metabolic, and quality of life traits will facilitate personalized precision interventions and the implementation of targeted public health policies.

## 1. Introduction

Chronic non-communicable diseases are a worldwide major public health burden [1]. Within these morbidities and accompanying increases in premature deaths, there are some associated lifestyles, where dietary habits, the practice of physical activity, and healthy attitudes play a crucial role in their development [2]. Current medical recommendations are focused on following a healthy balanced dietary pattern, regular practice of physical activity, and quitting smoking and alcoholic drinking as a series of good practices to preserve a steady state of health [3]. Indeed, nutrition is usually a relevant approach to determine better cardiometabolic health and to prevent the appearance of chronic non-communicable diseases including obesity, diabetes, and dyslipidemia [4].

Food intake data have been traditionally collected through different validated questionnaires, such as 24 h recalls, the Food Frequency Questionnaire (FFQ), or a detailed dietary history [5]. However, an issue with these questionnaires is that they depend on the patient’s subjective opinion/memory and the professional expertise of the researchers conducting the data analyses; moreover, several questionnaires need to be performed [6]. While it remains true that conventional methods appear useful for routine clinical practice, is imperative for health professionals to develop techniques with an integrated measurement of food and nutrient consumption or physical activity habits in contemporary studies [7]. The assessment of lifestyle includes physical activity based on validated questionnaires such as the International Physical Activity Questionnaire (IPAQ) [8] and quality of life, which can be determined by the 12-item Short Form Survey (SF-12) [9]. These data ensure accurate evaluation of lifestyle aspects and well-being and can benefit from strategies combining some of them.

Thus, the evaluation of health status requires a multidimensional approach based on numerous variables, making a simple evaluation difficult to integrate data from different sources in medical services and primary healthcare [10]. In this sense, it is important to consider precision nutrition instruments for lifestyle evaluation [11]. The use of computational analysis based on machine learning tools and statistical clustering methods allows the grouping of these variables to define a multidimensional patient profile [12]. In fact, in recent years there has been an increase in the development of nutritional indices based on computer algorithms that allow the population to be classified into specific subgroups [13]. These nutritional indices qualitatively classify each individual, taking into account relevant characteristics to create metabotypes [14]. In this sense, the development of screening instruments and scales, which help to simplify decision-making in clinical practice, is an important point of analysis for suitable epidemiological implementation. The validity of these scores and measurements depends on the accurate collection of personalized health information, physical activities, quality of life, and suitable health markers.

Currently, the study of nutritional indices makes it possible to quickly stratify population groups with similar metabolic and lifestyle characteristics [15]. The use of these tools by the health professional requires a prior training process to understand their functionality and applicability [16]. At the same time, the information derived from the use of these tools must be communicated effectively with an informative component disseminated to society and citizens [17].

The aims of this study are (a) to provide a comprehensive description of the lifestyle habits prevalent among a Mediterranean population and (b) to devise an efficient tool for swiftly categorizing individuals based on lifestyle variables to serve as a valuable resource for informing and facilitating the implementation of targeted public health policies and interventions.

## 2. Materials and Methods

### 2.1. Study Design

The PLENUFAR 7 initiative was part of a broader effort to educate health professionals on nutrition and lifestyle assessment matters in Spain. A cross-sectional observational study, sponsored by the General Council of Pharmacists (CONGRAL, Madrid, Spain), was unfolded in two distinct phases spanning January 2021 to May 2022. The initial phase centered on professional training and information gathering, involving the registration of interested pharmacists and the dissemination of educational materials. The subsequent phase focused on participant recruitment and disseminating information to the public, with accredited pharmacists executing the formal recruitment and filling out online questionnaires between March and May 2022. The project received approval from the IMDEA Food Ethics Committee (CEI IMD-Pi-051, Madrid, Spain).

### 2.2. Participants

A total of 5496 volunteers were recruited by the participating establishments. Prior to enrollment, comprehensive information regarding data protection and informed consent was given to active pharmacists. Inclusion criteria were (a) to be aged between 18 and 75 years and (b) to provide signed informed consent. On the other hand, exclusion criteria encompassed (1) pregnant or breastfeeding women, (2) inadequate proficiency in Spanish communication, (3) individuals with disabilities or impediments hindering questionnaire comprehension and completion, and (4) the voluntary decision to not participate after initiating the survey. Upon enrollment, participants were granted access to anonymized questionnaires for completion.

### 2.3. Data Collection

General health and lifestyle data were collected based on validated questionnaires [18]. The study also included dietary habits through a food frequency survey encompassing 19 food groups [19]. Adherence to the Mediterranean diet was estimated using MEDAS-17 p [20]. This validated 17-item questionnaire defines four categories of adherence: low (0–6 p), low-to-moderate (7–8 p), moderate-to-high (9–10 p), and high (11–17 p). Additionally, quality of life and health status information was derived from SF-12 [21], which is a subset of 12 items (0–100 p) from the SF-36, and physical activity was assessed by the International Physical Activity Questionnaire (IPAQ-SF), adapted for the Spanish population [8]. Missing data were not significant (less than 2%) since self-reported questionnaires have mandatory questions that are required in order to proceed with data collection.

### 2.4. Nutritional Indices

The analysis of nutritional indices was carried out using four a priori scores to measure the degree of quality of health in the participants based on validated scales. The first of these was the adapted Life Simple 7 (LS7) nutritional score, which uses the definition proposed by the American Heart Association of the seven most important predictors of heart health to achieve ideal cardiovascular health [22]. This score includes a total of seven factors, including four modifiable behaviors (non-smoking, healthy weight, healthy eating, and physical activity) and three biometric measures (blood pressure, cholesterol, and blood sugar). In turn, these factors are classified into three categories: ideal (2 points), intermediate (1 point), and poor (0 points). Because there was no quantitative information on blood pressure, dyslipidemia, and glycemia levels, a criterion of 0 points was established for participants who had hypertension, dyslipidemia, and hyperglycemia. Individuals without any prior circumstances were assigned a score of 2. Participants with ideal levels for all seven metrics were considered to have ideal cardiovascular health.

The second nutritional score was an adaptation of the former Mediterranean Lifestyle (MEDLIFE) index [23]. The MEDLIFE index was created following the principles of the Mediterranean Diet Pyramid [24] proposed by the Spanish Mediterranean Diet Foundation. The original score presented a total of 28 items. The scoring was adapted for some questions since it could not be interpolated from the entire PLENUFAR 7 questionnaire. Each of the derived items obtained a possible score of 0 (if not compliant) or 1 (if compliant).

The third performed metabolic/well-being computational score was the Healthy Heart Score (HHS) [25], based on a model for predicting the risk of cardiovascular disease (non-fatal myocardial infarction, fatal coronary artery, and ischemic stroke) that estimates the 20-year risk. It uses lifestyle factors developed within 2 US cohorts (HPFS and NHS) free of cardiovascular disease, diabetes mellitus, and cancer. This score includes the 9 factors that best estimate the risk of cardiovascular disease: current smoking; higher BMI; low physical activity; excessive or no alcohol consumption; low intake of fruits, vegetables, and fiber from cereals or nuts; and high consumption of sugary drinks or red/processed meats. A higher score (expressed in %) reflects a higher risk of cardiovascular disease (CVD).

The adapted score of the fourth nutritional index was the Mediterranean diet-related Healthy Lifestyle score (HLS), which combines lifestyle factors considered “optimal” versus “non-optimal”. The optimal criteria receive 1 point each, so the points obtained from the following five concepts are added to assess a score between 0 (non-optimal lifestyle) and 5 (most optimal style of all): on the Body Mass Index (BMI), normal (BMI  <  25 kg/m^2^) versus obese (BMI  ≥  25 kg/m^2^); good adherence (≥6 points) or poor adherence (<6 points) to the Mediterranean diet; adequate physical activity (>150 min/week) versus non-optimal activity (0 < 150 min/week); smoking habits between no smoking versus smoking/quitting smoking; and frequent alcohol consumption versus little/no consumption [26]. This HLS punctuation is categorized as poor (0–1), intermediate (2), and excellent (3–5).

### 2.5. Statistical Data

An exploratory factor analysis was applied to 91 variables (Supplementary Materials, Table S1) was carried out in the STATA software (v18, StataCorp LLC, College Station, TX, USA). The grouping scheme was based on the similarity of the variable profile of each of the questions and the weight provided by the model. To identify reduced dimensionality and obtain latent factors of data, an exploratory factor analysis using the principal components method was performed [27]. To determine the number of factors to retain, the Scree test was carried out with values >1 and the interpretability of the factors [28]. The factors were rotated with an orthogonal rotation procedure (promax rotation) so that uncorrelated factors were derived and the results were easier to interpret. For each participant, a factor score was calculated from the factor obtained in the final analysis. These scores were used to perform a hierarchical cluster analysis to discern different patterns in the population (metabotypes) according to the statistical weight that each latent variable exerted for each subject.

To minimize variance within resulting clusters and to create clusters that are compact and well separated, clustering analysis was performed using Ward’s Linkage Cluster method with the utilization of Euclidean distance [29] with the command cluster wardslinkage in STATA. Ward’s linkage method, known for its sustainable ability to create compact and spherical clusters, employs the Euclidean distance metric to calculate distances between all pairs of observations based on the factor variables [30]. This Euclidean distance calculation was pivotal in constructing the distance matrix, which represents the pairwise distances between observations. Subsequently, the Ward’s method aimed to minimize the sum of squared differences within each cluster by merging pairs of clusters with the smallest Euclidean distances [29]. The clustering process was guided by the objective of creating homogeneous clusters with minimal within-cluster variance, a task facilitated by the sensitivity of Ward’s method to outliers. Additionally, the hierarchical nature of Ward’s linkage provided a comprehensive view of the clustering process, which was visually represented by dendrograms [31] using the cluster tree command in STATA. In the construction of the dendrograms, the Euclidean distance squared was utilized as the distance measure between clusters on the vertical axis. The heights of the fusion points in the dendrograms corresponded to the squared Euclidean distances between the merging clusters, offering insights into the clustering structure. To determine the optimal number of clusters, the cut-off point was identified using Calinski and Harabasz’s pseudo-F index (cluster stop command in STATA) [32], which evaluates the clustering quality based on the ratio of between-cluster dispersion to within-cluster dispersion [33]. This index allowed for the visualization of the optimal number of clusters through dendrograms, aiding in the interpretation and selection of the most suitable clustering solution. The process resulted in the identification of distinct clusters, providing a valuable framework for further analysis and interpretation of the dataset.

To develop a classification tool, the beta coefficients (ß) of the variables comprising the computational algorithm of metabotypes were obtained by applying multiple regression between the clusters and the most relevant variables for the computational algorithm (age, sex, occupation, education, smoking, cigarettes per day, cohabitation, nap, weekday sleep hours, weekend sleep hours, obesity, diabetes, arterial hypertension, dyslipidemia, BMI, self-perception of weight loss, self-perception of weight gain, snacking, number of meals per day, frequency of table salt use, MEDAS-17 p score, self-perception of health, feeling down or sad, and hours of intense and moderate physical activity). Model fit was assessed through examination of the R-squared value and adjusted R-squared value, indicating the degree of adjustment of the prediction to the data. Furthermore, the presence of multicollinearity among the predictor variables was evaluated by calculating variance inflation factors (VIFs) for all independent variables. VIF values below the commonly accepted threshold of 4 or 5 suggested no significant multicollinearity issues, affirming the reliability of the estimated regression coefficients. These methodological steps ensure a solid foundation for the development and interpretation of the metabotype classification tool.

Subsequently, a random forest model, with value for categorization and predictive purposes was used (rforest command). This model is a commonly used machine learning algorithm generated as described elsewhere [34], which aggregates the outputs of multiple decision trees to generate a single result, being ease of use and flexibility have led to widespread adoption, as it addresses both classification and regression tasks. The model considered the main variables of the computation algorithm such as age, sex, occupation, education, smoking, cigarettes a day, cohabitation, nap, sleeping hours during the week, sleeping hours on weekends, obesity, diabetes, high blood pressure, dyslipidemia, BMI, self-perception of losing weight, self-perception of gaining weight, snacking, number of meals per day, frequency of use of table salt, MEDAS-17 p score, self-perception of health, feeling discouraged or sad, and hours of intense and moderate physical activity were taken into account for the model. The model used a total of 500 iterations to establish participants’ probabilities of being classified into other metabotype groups. The importance matrices were reviewed to understand which variables have the greatest impact on the classification of participants into the different metabolic groups.

## 3. Results

### 3.1. General Data

PLENUFAR 7 enrolled 5496 participants, with a predominance of women (*n* = 3363) compared to men (*n* = 2105). On average, the volunteers were 47.4 years old with a BMI of 25.2 kg/m^2^. According to the World Health Organization (WHO), overweight is characterized by a BMI ranging from 25.0 to 29.9 kg/m^2^, whereas type I obesity is delineated by a BMI falling between 30.0 and 34.9 kg/m^2^ [35]. A total of 33.1% of participants were classified as overweight, and 13.4% of participants had at least type I obesity. Breaking it down by gender, 41% of men were classified as overweight and 16.7% had at least type I obesity; while 28.2% of women were classified as overweight, and 11.4% had at least type I obesity. Additional information on dietary habits, lifestyle variables, quality of life, and physical activities is given in Table 1.

### 3.2. Dietary Intake Assessment

Comprehensive data regarding the frequency of food consumption can be found in Table 2. A total of 51.8% of the volunteers abstained from fats other than extra virgin olive oil. Moreover, a notable portion of participants (48.5%) did not incorporate whole grains into their regular diet. In terms of frequency, fatty meat consumption appears intermittent monthly, while legumes are typically consumed sparingly, with 62.7% opting for only 1–2 servings per week, and 44.9% consuming 3–4 eggs weekly. Lean meat intake ranges from 1–4 times weekly, whereas whitefish and bluefish are typically consumed 1–2 times weekly. In daily consumption, emphasis is placed on 1–2 servings of semi-skimmed dairy products (44.8%), vegetables (41.9%), fruits (47%), and notably, extra virgin olive oil (63.3%). The sole item consumed more than five times daily is water (44.5%).

### 3.3. Adherence to Mediterranean Diet Pattern

MEDAS results can be found in Table 3. Percentages of 19.2%, 9.7%, 24.8%, and 46.4% of the subjects exhibited low, low-medium, medium-high, and high adherence to the Mediterranean diet, respectively. The lowest punctuations (<50%) were displayed by fruit, legumes, fish, nuts, and whole cereals consumption. In broad terms, olive oil was the main source of fat from the diet (89.5%), with moderate use (ranging from never/almost never to 1–2 times per week) of unhealthy fats (butter, margarine, or cream). Regarding plant-based foods, 63.2% of the participants consumed ≤2 pieces of fruit per day; 69.9% consumed ≤2 servings of legumes per week; and proportionally, there was a balance between those who incorporated 0–1 servings per day (48.8%) and ≥2 servings per day (51.2%) of vegetables. As for protein-rich foods, 60.6% ate ≤2 servings of fish per week; 62.4% consumed ≤2 servings of nuts per week. Additionally, 77.7% consumed ≤4 servings of cereals and whole foods (bread, rice, and pasta) per week, and 88.5% consumed wine moderately (men between two and three glasses per day; women between one and two glasses per day). The preceding findings align with those observed in the FFQ.

### 3.4. Physical Activity Data

In the PLENUFAR 7 study, the staple physical activity among participants was walking (Table 4), with 48% engaging in this activity for durations ranging between 30 and 60 min (37.6% and 38%, respectively). Over half of the population engaged in intense physical activity at least once a week (55.9%), with a more pronounced inclination toward moderate activities (71%). The typical sitting time fell within the range of 2 to 7 h for 65.6% of individuals.

### 3.5. Quality of Life Results

Findings from the SF-12 questionnaire regarding quality of life are detailed in Table 5. Approximately half of the volunteers perceive their health positively (49.9%), reporting no mobility or routine task limitations. In the 30 days preceding the survey, most volunteers did not experience any physical or emotional issues (>80%), although a small portion of the population mentioned experiencing pain during that period (37.9%). In terms of mental health, the population generally reported a good overall mood. However, the impact of emotional states on social interactions is noteworthy, with 56.7% of the population reporting experiencing periods of social isolation at some point.

### 3.6. Nutritional Indices and Metabotype Computational Algorithm

The variables derived from the preceding data were consumed to compute the nutritional indices LS7, MEDLIFE, HHS, and HLS. Moderate scores were observed for LS7 (7.9 ± 1.7), MEDLIFE (9.3 ± 2.6), and HLS (2.4 ± 0.8), whereas HHS indicated a low percentage for cardiovascular risk (12 ± 21.5). Then, an equation was created to estimate the classification of each metabotype derived from demographic information and the former nutritional indices data. Information about the equation and the beta coefficients of each variable (superscripts) are given in the Supplementary Materials (Table S2):

Metabotype estimation = [– 0.0768] + (age × 0.0084) + (sex × n^1^) + (occupation × n^2^) + (education × n^3^) + (smoking × n^4^) + (cigarettes per day × n^5^) + (cohabitation × n^6^) + (hours of nap during the week × n^7^) + (hours of weekend nap × n^8^) + (weekday sleep times × n^9^) + (weekend sleep times × n^10^) + (obesity × n^11^) + (DMII × n^12^) + (hypertension × n^13^) + (dyslipidemia × n^14^) + (BMI × 0.0446041) + (self-perception of losing weight × n^15^) + (self-perception of gaining weight × n^16^) + (snacking × n^17^) + (number of meals per day × n^18^) + (use table salt × n^19^) + (MEDAS-17 p score × 0.1531) + (self-perception of health × n^20^) + (disheartened or sad × n^21^) + (hours of intense physical activity × 0.0471) + (hours of active moderate physical × [−0.0221])

**Figure 1:**
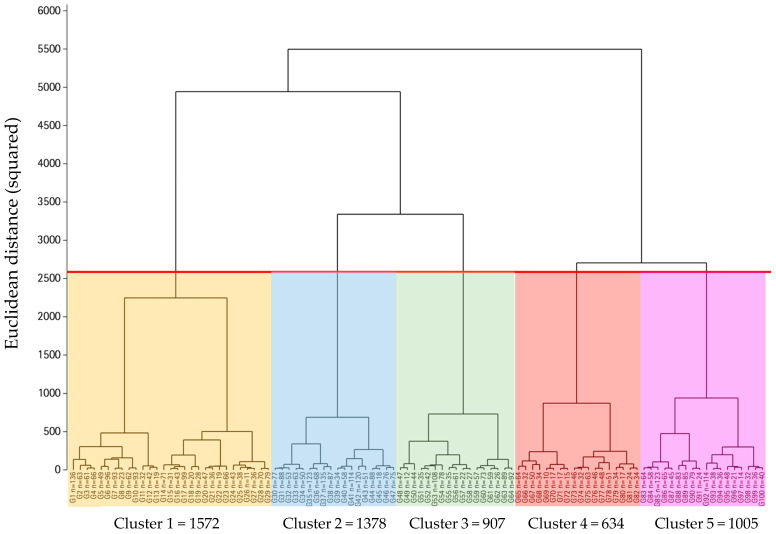
Obtained dendrogram of cluster analysis derived from the PLENUFAR 7 lifestyle, diet, physical activity, quality of life, and nutritional indices results.

For this purpose, a hierarchical cluster analysis using Ward’s method [36] was developed to generate a dendrogram that depicts the clustering of the 5496 observations into five clusters (Figure 1): Westernized Millennial (Metabotype 1, *n* = 1572), healthy (Metabotype 2, *n* = 1378), active Mediterranean (Metabotype 3, *n* = 907), dysmetabolic/pre-morbid (Metabotype 4, *n* = 634), and metabolically vulnerable/pro-morbid (Metabotype 5, *n* = 1005). The squared Euclidean distance was employed as a dissimilarity measure to assess the similarity between observations. The vertical axis represents the squared Euclidean distance between observations or groups of observations. A higher value indicates lower similarity. The horizontal axis shows the observations or groups undergoing merging. Horizontal lines represent the joining of groups, with their height indicating the distance at which they merged. At the selected cut-off level, five distinct clusters were identified, differentiated by colors. The cut-off points were set as follows: 1–1.49 (metabotype 1), 1.5–2.49 (metabotype 2), 2.5–3.49 (metabotype 3), 3.5–4.49 (metabotype 4), and ≥4.5/metabotype 5). The probability of being classified into other metabotype groups is shown in the Supplementary Materials (Table S3).

### 3.7. Metabotype Characterization

The description of metabotypes is presented in Table 6. All the variables were included in the exploratory factor analysis process. However, items collected in the characteristics of food (FFQ), disease, and adherence to the Mediterranean diet (MEDAS-17 p) were only used to improve the representation of certain factors of the model. Metabotypes 1 and 2 share the absence of cardiovascular pathologies, good sleep quality, normal BMI, and significant physical activity. The differences between these clusters lie in the excessive salt intake and low adherence to the Mediterranean diet, coupled with a negative perception of mental well-being in metabotype 1, whereas metabotype 2 emerges as the healthiest option. Metabotypes 3, 4, and 5 exhibit various chronic non-communicable diseases (such as diabetes, obesity, and cardiovascular disease), overweight, and a tendency to nap regularly. However, the dietary habits and physical activity levels described in metabotype 4 deviate further from the Mediterranean diet and WHO recommendations compared to metabotypes 3 and 5. Metabotypes 4 and 5 also show a higher proportion of retired individuals, with less rest during the week and reduced quality of life, reflected in lower SF-12 scores. Overall, metabotype 4 is considered the least favorable and associated with the highest cardiometabolic risk.

### 3.8. Metabotype Characterization

**Figure 2:**
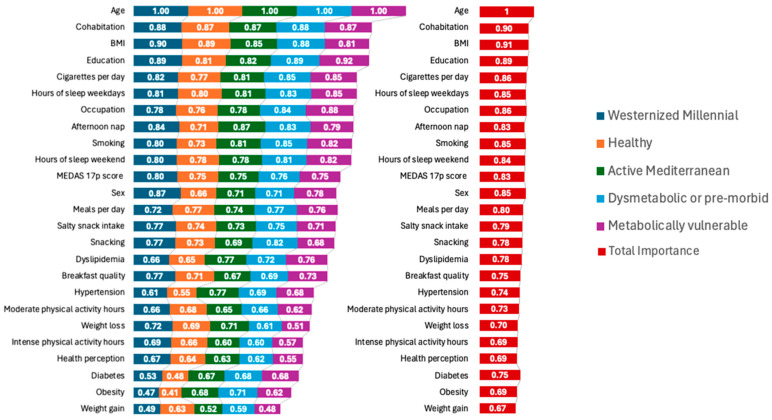
Stratified feature importance analysis raised using a random forest model in PLENUFAR 7.

The total importance scores suggest that several variables collectively play significant roles in determining the metabolic profiles and health statuses within PLENUFAR 7 (Figure 2). Age, a fundamental factor, was uniformly weighted across all subgroups, indicating its consistent relevance. Cohabitation emerged as a notable contributor across all metabolic categories, emphasizing the potential influence of shared living arrangements on health behaviors and outcomes. This finding suggests that individuals’ living situations may impact their metabolic vulnerabilities and pre-morbid conditions consistently. BMI demonstrated high importance scores across all categories, especially within the dysmetabolic/pre-morbid and metabolically vulnerable/pro-morbid groups, underlining a critical role in these individuals’ health profiles. Education levels also displayed consistent importance, indicating its association with various metabolic states among Westernized Millennials. Furthermore, lifestyle factors such as smoking, sleep patterns, and physical activity showed nuanced importance scores. Smoking habits were notably relevant, particularly within the dysmetabolic/pre-morbid and metabolically vulnerable/pro-morbid groups, suggesting its potential exacerbating effects on metabolic health. Sleep patterns, especially weekday sleep duration, appeared consistently influential across all groups, indicating its role in metabolic vulnerability. Interestingly, Mediterranean diet adherence, as represented by the MEDAS-17 p score, demonstrated notable importance, particularly within the Westernized Millennial and dysmetabolic/pre-morbid groups. This underscores the potential benefits of a Mediterranean diet in mitigating metabolic risks within this demographic. Moreover, gender differences were evident, with sex showing varying importance across the different metabolic profiles. These findings suggest that gender-specific health considerations may be pertinent in understanding the metabolic vulnerabilities of Westernized Millennials.

## 4. Discussion

Ensuring safe lifestyles is essential for maintaining optimal health and preventing the onset of chronic non-communicable diseases at both individual and population levels, which should be based on objective and quantitative determinations [37]. Previously, the analysis of a longitudinal cohort derived from the UK Biobank reported that a healthier lifestyle was associated with up to 6.3 more years of life for men and 7.6 years for women [38]. Key influencers in this regard include nutrition and physical activity, both of which are modifiable factors and can be tailored in a personalized manner. Presently, there is a growing societal emphasis on adhering to a healthy diet and engaging in physical activity to enhance overall well-being [39]. Within this context, healthcare professionals may play a crucial role in disseminating the latest scientific insights pertaining to diet and exercise to educate the public about adopting healthy lifestyle habits [16]. The former approach and up-to-date training from reputable sources or accredited organizations are essential to effectively execute precision public health interventions. Precision nutrition aims to incorporate advanced information to develop holistic strategies suitable for widespread application and integration of several features with objective values [40]. This field combines numerous variables specific to each individual, which are determined through bioinformatic analysis techniques [41]. The analyzed data enabled the calculation of scores for objectively quantifying the global influence of exposure, metabolic, and physical factors on an individual’s metabolic status. By employing decision algorithms based on machine learning, individuals can be qualitatively categorized based on criteria related to nutrition, lifestyle, and metabolic well-being, leading to the definition of metabotypes or nutritypes [42]. It is imperative for healthcare professionals to know and validate the tools provided by precision nutrition in order to effectively apply and communicate them to the population.

Previously, several cohorts have been designed to provide nutritional and lifestyle features associated with health outcomes in Spain. For instance, the ANIBES project conducted a deep study on food consumption based on sex and age groups [43]. This information was useful for establishing a starting point about dietary patterns’ influence on health. The generation of healthy scores related to lifestyle and well-being was also encompassed by the SUN project [44]. In Europe, the stratification of variables derived from a prognostic tool was used to identify differences in the EPIC study population [45]; moreover, the use of clustering analysis to define cardiometabolic risk associated with overweight has been reported since 2008 in the NHANES cohort [46]. These cohorts have used to a greater or lesser extent instruments related to clustering and classification of subgroups, which demonstrate the value and benefit of this information, despite differences in the cohorts. In any case, results can be ascribed to temporal settings, culture, using questionnaires, ethnic groups, purposes, and scenarios, thus standardization of the developed tools could be complex.

PLENUFAR 7 has achieved an updated characterization of the Spanish population by incorporating lifestyle variables. Through advanced machine learning techniques on the acquired data, a classification instrument has been developed. The use of this innovative tool by healthcare professionals could streamline the provision of more tailored dietary and lifestyle guidance, thereby enhancing overall health outcomes. Since the turn of the current century, especially during the COVID-19 pandemic, the Mediterranean dietary pattern has undergone shifts that have changed behavioral and dietary habits [47]. However, these modifications have not sharply decreased adherence to the traditional Mediterranean diet, allowing the Spanish population to maintain this dietary pattern in the current situation [48]. The benefits of the Mediterranean diet in preventing chronic pathologies have been widely studied and demonstrated [49,50,51,52]. Furthermore, a healthy dietary pattern is also related to a lower risk of suffering from DMII and CVD as stated by the study of the UK Biobank [53]. In addition, the Framingham Heart Study pinpointed the need to implement proteomic and metabolomic data to unveil mechanisms mediating diet-related disease in healthy dietary patterns [54]. Nevertheless, the data gathered from PLENUFAR 7 indicate the need to strengthen the existing dietary pattern to enhance adherence among the surveyed population, even though deeper analysis including omics data should be conducted abroad in the future.

In the EPIC Potsdam study, factors such as vegetables (raw and cooked), meat, sauces, refined grains, and fat foods (high-fat cheeses, butter, margarine, desserts, and pastries) determined the composition of a dietary pattern [55]. Later, in this Spanish cohort, a notable decrease in overall mortality rates and lower risk of CVD was determined by adhering to a Mediterranean diet abundant in olive oil [56]. A prior study conducted within the Spanish population revealed that the consumption levels of fruits, cereals, legumes, fish and seafood, red meat, and carbonated beverages were relevant factors influencing adherence to the Mediterranean pattern [57]. While the present study similarly observed low consumption levels of legumes, fruits, and fish, noteworthy factors contributing to adherence within the surveyed population included elevated consumption of pastries, olive oil, and wine. However, fish consumption is a main issue to pinpoint since the ANIBES study showed that the intake of omega-3 and omega-6 polyunsaturated fatty acids was significantly low, necessitating the search for alternative sources to avoid potential population deficits [58]. On the other hand, although vegetable consumption levels as measured by both the MEDAS and the FFQ were not excessively low, there is room for improvement. Various factors such as female gender, completion of university studies, and age are associated with greater vegetable consumption, whereas being overweight typically correlates negatively with vegetable intake [59]. These findings are likely generalizable to the broader population of PLENUFAR 7, given the higher proportion of female respondents. However, it is plausible that the presence of overweight men might have somewhat reduced the average vegetable consumption among participants. Nevertheless, efforts to enhance daily vegetable intake should remain a focal point for improvement campaigns led by health professionals, despite the possible lack of direct impact on adherence to the Mediterranean diet [60]. Other data derived from the UK biobank pointed out not only the importance of the dietary pattern, but also the associations between carbohydrates and fat intake and the risk of suffering from CVD, so the analysis of these macronutrients in diet is another factor to consider in the future [53,61]. Healthcare professionals should integrate advanced dietary data to develop tailored advice based on a personalized approach [62].

Conversely, the physical activity levels of the population underwent notable changes during the COVID-19 pandemic [63]. Despite the benefits of a balanced dietary pattern, the effects of reduction are only observed if it is accompanied by physical activity [64]. The World Health Organization (WHO) recommends between 150–300 min/week of moderate activity, 75–150 min/week of vigorous or intense activity, or an equivalent combination of both, to maintain an active lifestyle [65]. The PLENUFAR 6 study, the predecessor of the current work, showed that around 50% of the participants carried out vigorous physical activity, and 23.4% implemented moderate activity, like those obtained in the present study [66]. Additionally, the SUN project described that sedentary attitudes related to computer use were associated with more depressive states in the population [67]. It is obvious that health policies must try to integrate the practice of physical activity in the Spanish population, to reduce the cardiovascular risk that is increased by following a sedentary lifestyle and potential mental illness [68].

Regarding the results on quality of life, the information extracted from the project shows a positive level in the study participants. A total of 50% of the PLENUFAR 7 participants perceive their health as good through the SF-12 questionnaire, indicating an average score of 64.13/100 on physical and mental health. Previously, a study was carried out in the Spanish population on the well-being associated with healthy lifestyle habits. The results showed that the perception of quality of life as well as purchasing power were the factors that most influenced quality of life, together with a healthy diet and a good social environment [69]. The SUN Study also highlighted that adherence to the Mediterranean diet or pro-vegetarian food patterns, physical activity, and sleep are linked to a higher quality of life [70]. Thus, it seems the more plant-based diet adherence is practiced, the better the expected results over this lifestyle dimension.

Nutritional indices are being increasingly implemented in cohort studies due to their potential to integrate different variables associated primarily with CVD. The PREDIMED study used this score to evaluate the incidence of major cardiovascular events in the Spanish population, describing a lower risk when LS7 values were higher than 9 p including as many metrics as possible [71]. In the NutrIMDEA Web-Based Study, a 4.48 ± 1.1 p was obtained, being lower than the LS7 value of the present study (7.9 out of 14 p) [72]. These results imply a certain cardiovascular risk in the PLENUFAR 7 population associated with lifestyle using this nutritional index. On the other hand, through the MEDLIFE nutritional score, the risk of mortality from CVD in the population can be determined. Previously, it was described that high scores were associated with lower HOMA-IR and highly sensitive C-reactive protein, which is a useful scale to determine metabolic syndrome [73]. In a different patient cohort from the CORDIOPREV study, it was found that for each additional point on the MEDLIFE scale, the risk of developing metabolic syndrome decreased by 24%, with a 21% chance of reversing the condition [74]. The inverse relation between CVD risk and MEDLIFE punctuation was also highlighted in the SUN cohort [75]. Despite the population’s moderate adherence to the Mediterranean diet in PLENUFAR 7, the reality is that low consumption of plant-based products may result in a lower score on the MEDLIFE scale (<12 points). Therefore, it is necessary to communicate this tool to the population to increase awareness about adhering correctly to the Mediterranean pattern.

Additionally, HLS was also determined in the NutrIMDEA study with a 2.37 ± 0.8 average value [72], which is similar to the results obtained in PLENUFAR 7. Regarding the HHS, it serves as a valuable tool in predicting premature vascular risk, as evidenced by the CARDIA study [76] as well as the ENRICA study [77]. Due to its ease of interpretation, user-friendly tools such as the HHS can be effectively integrated into health education programs for individuals at risk of CVD [78].

Concerning the use of equations to analyze cardiometabolic risk, the Framingham Study previously reported the estimation of CVD using an estimation approach [79]. Thus, using advanced tools based on machine learning can be applied to health sciences, mainly through clustering models [80]. PLENUFAR 7 investigation has important clinical implications that can contribute to precision public health by focusing messages on specific clusters to contribute to the implementation of epidemiological policies as well as to promote specific health actions. In the work developed by Higuera-Gómez et al., an obesogenic score was reported including lifestyle and life quality items, which can be used in the general population [81]. On the other hand, the clusterization system may provide valuable support in clinical care and nutrition guidance for both people who are healthy and those with disease as diagnostic and prognostic tools for precision prescription display on metabolic traits, lifestyle factors, and phenotypical features. For instance, factorial analysis was performed previously to classify subjects depending on dietary patterns (proto-omnivorous versus plant-based diets) and health outcomes [82]. Additionally, cardiovascular risk was assessed using cluster analysis in metabolic syndrome patients including phenotypical and clinical variables habitually collected during health check-ups [83]. Moreover, this approach could implement specific interventions in chronic diseases such as diabetes [84] or weight loss, such as the PREVIEW study [85]. The use of advanced data analysis techniques involves a complex but much deeper interpretation of the data extracted from an individual. The integration of qualitative and quantitative information means better decision-making that can be applied to dietary and lifestyle advice provided by the health professional [86]. In the EPIC study, a cluster analysis was carried out in which two dietary profiles were determined: a plant-based pattern and another characterized by a high intake of sweets and fatty foods, facilitating the categorization of the population with respect to mortality rates derived from the consumed diet [87]. The use of this technique has also been used to determine the risk of suffering from cardiometabolic diseases in a young population [88].

### Study Limitations and Strengths

The participation of the Spanish population in each of the establishments was not homogeneous due to the inclusion and exclusion criteria allowing the recruitment of a large number of volunteers with different characteristics. However, the sampling stratification allows the results to be extrapolated to the general population level given the number of participants (*n* = 5496) and the wide inclusion criteria set up. Moreover, the health professional in charge of the interviews could generate bias when collecting the data by forming subjective interpretations of the answers provided by the participants. The use of closed questionnaires can lead to limited information on the population, as in the case of MEDAS-17 p, in which foods such as eggs or dairy products were not included, which was overcome using various questionnaires. Additionally, self-reported data could generate some bias related to sedentary time, energy intake from foods, and physical activity, although good reliability is usually described in this assessment methodology [89,90,91].

On the other hand, information on the relationship between physical activity and body composition could generate very useful information to establish correlations between diet and food; therefore, adding the study of more anthropometric and body composition data in the future would mean an improvement in the interpretation of the data obtained. Healthcare workers such as nurses and preventive medical services can benefit from the use of new instruments based on precision nutrition, allowing better screening of patients.

## 5. Conclusions

PLENUFAR 7 has outlined key lifestyle factors among a diverse sample of the Spanish population. Healthcare professionals should prioritize promoting the Mediterranean diet, encouraging moderation in alcohol consumption, and advocating for increased consumption of plant-based foods. Additionally, it is crucial to underscore the importance of regular physical activity, whether through walking or tailored exercises that suit individual needs.

By utilizing a clustering model, the population has been categorized into five metabotypes (Westernized Millennial, healthy, active Mediterranean, dysmetabolic/pre-morbid, and metabolically vulnerable/pro-morbid) with the potential for precise medical classification. Integrating metabotypes into primary care could streamline the identification of unhealthy lifestyles, enhancing healthcare providers’ ability to tailor patient care effectively.

Healthcare professionals need to undergo training to effectively utilize these new, objective tools within the realm of precision public health. Simultaneously, raising awareness among the public about metabotype classification is essential. Overall, the PLENUFAR 7 project successfully categorizes the population into metabotypes, enabling personalized interventions related to nutrition and health, as well as providing insights into well-being-related habits and attitudes.

## Figures and Tables

**Table 1:** Anthropometric and lifestyle information derived from the PLENUFAR 7 participants.

Variables	Frequency	Men	Women	*p* ^1^
Age (years)		47.63 ± 17.81	47.24 ± 17.06	0.422
Weight (kg)		80.87 ± 13.23	65.05 ± 12.03	<0.001
BMI (kg/m^2^)		26.32 ± 4.11	24.48 ± 4.53	<0.001
MEDAS (0–17 p)		8.58 ± 3.04	9.43 ± 2.87	<0.001
IPAQ (kcal/week)		3442 ± 4168	1746 ± 2095	<0.001
SF-12 (PD) (0–100 p)		33.3 ± 6.1	33.6 ± 6.1	0.073
SF-12 (MD) (0–100 p)		30.7 ± 6.7	30.6 ± 6.7	0.578
Number of meals/day (%)	<2	6.60	4.25	0.001
	3–5	92.16	94.65	
	>6	1.24	1.10	
Number of glasses of water/day (%)	<4	38.37	39.73	0.066
	4–7	28.27	29.79	
	>7	33.17	30.48	
Hours of sleep per night/day (M-F) (%)	<5 h	4.37	3.95	0.003
	5–9 h	91.07	91.02	
	>9 h	4.57	5.03	
Hours of sleep per night/day (S-S) (%)	<5 h	3.23	3.27	0.031
	5–9 h	77.29	74.60	
	>9 h	19.48	22.13	

Data are presented as mean ± standard deviation (SD), except for those related to eating behavior and resting, which are shown in percentages (%). IPAQ: International Physical Activity Questionnaire; MD: mental domain; MEDAS: Adherence to Mediterranean diet; M-F: Monday to Friday; PD: physical domain; and S-S: Saturday to Sunday. ^1^ The *p*-values were obtained using linear regression for continuous variables and multinomial logistic regression for categorical data adjusting for sex.

**Table 2:** Data based on responses to the Food Frequency Questionnaire (FFQ) including 19 foods characteristic of the Mediterranean diet.

Food Group	Never/ Almost Never		Times Per Month		Times Per Week						Times Per Day						*p* ^1^
			1–3		1–2		3–4		5–6		1–2		3–4		>5		
	M	W	M	W	M	W	M	W	M	W	M	W	M	W	M	W	
Whole daily	37.5	46.3	6.8	7.1	11.5	12.2	10.0	8.9	3.5	2.4	27.7	20.6	2.6	2.2	0.4	0.3	<0.001
Semi-skimmed Dairy	31.7	23.0	3.9	3.8	6.2	5.4	7.7	8.6	3.8	4.6	41.3	47.3	4.5	6.3	0.9	1.0	<0.001
Eggs	1.1	1.6	3.1	3.6	30.9	35.2	44.4	45.3	11.6	8.4	7.6	5.3	1.1	0.6	0.2	0.1	0.103
Lean meats	2.7	5.7	5.1	6.3	36.4	39.4	39.3	37.2	7.4	6.0	8.4	5.0	0.5	0.3	0.2	0.1	<0.001
Fatty meats	15.7	26.3	17.2	22.4	47.7	39.7	15.0	8.7	1.8	1.1	2.4	1.6	0.2	0.1	0.0	0.0	<0.001
Whitefish	9.2	7.7	16.0	12.5	59.3	59.4	12.9	17.7	1.2	1.6	1.3	1.0	0.1	0.2	0.1	0.0	0.051
Bluefish	12.7	10.5	21.0	18.5	55.9	59.8	8.7	9.4	1.1	0.7	0.6	1.0	0.0	0.1	0.6	0.0	0.010
Vegetables	4.0	1.6	3.0	1.4	17.2	10.8	24.9	22.0	10.0	11.8	36.6	45.4	3.7	5.8	0.6	1.3	<0.001
Fruits	5.7	3.5	2.0	1.7	6.6	5.2	11.2	10.3	6.2	6.5	44.7	48.7	19.7	21.8	4.0	2.4	<0.001
Nuts	24.3	22.2	13.8	13.0	22.9	22.8	13.4	14.0	3.7	3.8	19.7	22.8	1.4	1.1	0.7	0.5	0.064
Legumes	3.9	4.0	8.8	8.8	61.5	63.6	22.1	20.1	1.6	1.4	2.0	1.8	0.1	0.3	0.0	0.1	0.785
Olive oil	2.2	1.9	1.0	0.7	3.1	2.7	7.0	5.9	6.8	5.1	62.4	64.3	14.2	16.6	3.2	2.9	0.47
Other fats	50.6	52.6	13.7	13.7	19.2	17.4	7.0	6.4	1.3	1.5	7.5	8.1	0.4	0.3	0.3	0.0	0.133
Refined grains	37.3	40.4	7.2	7.9	15.4	17.1	10.4	9.2	3.1	2.5	22.1	20.0	4.0	2.7	0.3	0.3	0.026
Whole grains	56.9	43.3	6.9	8.1	11.7	13.9	6.1	8.0	2.2	2.6	15.1	22.6	1.0	1.2	0.1	0.3	<0.001
Processed bakery goods	38.5	43.8	14.4	16.5	22.3	20.6	10.5	8.1	2.3	1.7	11.5	8.9	0.4	0.4	0.1	0.2	<0.001
Sugars	35.4	41.5	8.5	8.6	11.8	13.5	8.4	6.9	2.8	2.4	30.4	25.1	2.1	1.7	0.6	0.4	<0.001
Alcohol	25.2	44.6	13.4	14.1	26.7	27.7	11.2	6.1	4.2	1.5	16.5	5.6	2.5	0.4	0.3	0.1	<0.001
Water	0.9	1.0	0.3	0.3	0.5	0.5	3.1	2.9	2.4	2.5	17.3	14.9	32.8	32.4	42.7	45.7	0.855

Data are presented as percentages (%). The Food Frequency Questionnaire (FFQ) allows for the estimation of the overall quality of the diet. ^1^ The *p*-values were obtained using multinomial logistic regression for categorical data adjusting for sex.

**Table 3:** Adherence to the Mediterranean diet of the Spanish population recruited in PLENUFAR 7 was measured through the validated MEDAS questionnaire (17 p).

Question	Positive Scale (+1 p)	Percentages (%)			
		Total	Men	Women	*p* ^1^
1. Use of extra virgin olive oil	Yes	89.5	88.9	90.1	0.180
2. Servings of vegetables/day	≥2 servings	51.2	45.9	54.3	<0.001
3. Pieces of fruit/day	≥3 pieces	36.8	35.6	37.4	0.0166
4. Servings of red meat, processed meats, or sausages/week	0–1 serving	50.0	41.5	55.4	<0.001
5. Servings of butter, margarine, or cream/week	<1 serving	83.3	82.4	84.0	0.134
6. Consumption of sugary drinks/week	<1 drink	73.8	68.1	77.5	<0.001
7. Servings of legumes/week	≥3 servings	30.1	30.4	29.7	0.626
8. Servings of fish or seafood/week	≥3 servings	39.4	36.3	41.3	<0.001
9. Pastry consumption/week	≤2 servings	69.0	65.2	71.3	<0.001
10. Nut consumption/week	≥3 servings	37.6	35.8	38.6	0.041
11. Greater consumption of white meat compared to red meat	Yes	76.8	69.2	81.7	<0.001
12. Dishes seasoned with sofrito/week	≥2 times	63.7	65.7	62.4	0.016
13. Consumption of sugary drinks	No	62.2	56.7	65.7	<0.001
14. Servings of white bread/day	0–1 time	59.9	51.2	65.3	<0.001
15. Servings of cereals and whole grain foods/week	≥5 servings	22.3	20.3	23.2	0.012
16. Servings of refined bread, rice, and/or pasta/week	≤2 servings	54.0	48.0	57.7	<0.001
17. Wine consumption	Yes	88.5	83.2	92.0	<0.001

^1^ The *p*-values were obtained using multinomial logistic regression for categorical data adjusting for sex.

**Table 4:** Performance of physical activity (intense, moderate, or walking) and frequency by PLENUFAR 7 participants derived from the Physical Activity Questionnaire (IPAQ).

Type of Physical Activity		0 d (%)	1 d (%)	2 d (%)	3 d (%)	4 d (%)	5 d (%)	>5 d (%)
Intense	**Frequency**	44.1	10.5	12.9	12.8	7.5	6.1	6.2
Moderate		29.0	13.3	16.3	13.7	7.6	7.9	12.2
Walking (>10′)		8.0	4.9	8.3	10.2	8.2	12.5	48.0
		**1–30′**	**30–60′**	**1–2 h**	**2–3 h**	**>3 h**		
Intense	**Timing**	52.8	25.1	16.6	3.1	2.3	n.a	n.a
Moderate		50.1	29.3	15.0	2.9	2.8	n.a	n.a
Walking (>10′)		37.6	38.0	18.0	3.6	2.8	n.a	n.a
		**1–2 h**	**2–4 h**	**5–7 h**	**8–10 h**	**>10 h**		
Sitting time	**Timing**	13.8	32.1	33.5	15.7	4.9	n.a	n.a

Physical activity METs (minutes/week): total: 1922.6; male: 2458.4; and female: 1576.6. Intense physical activity (minutes/week): total: 43.7; male: 53.8; and female: 37.2. Moderate physical activity (minutes/week): total: 44.3; male: 50.7; and female: 40.2. Walking (hours/week): total: 3.8; male: 3.8; and female: 3.7. n.a: not applicable.

**Table 5:** The quality of life assessment of the PLENUFAR 7 participants was carried out using the validated SF-12 questionnaire, including both physical and emotional health dimensions.

Question	Poor	Fair	Good	V. Good	Excellent	
1. How would you rate your health compared to people of your age?	2.4	17.2	49.9	23.5	6.9	
	No, it does not limit me at all		Yes, it limits me a little		Yes, it limits me	
2. Does your current health limit you from moderate efforts, such as moving a table, vacuuming, bowling, or walking for more than an hour?	80.6		14.0		5.3	
3. Does your current health limit you from climbing several flights of stairs?	75.3		17.2		7.4	
	No			Yes		
4. During the past 4 weeks, have you been limited to doing less than you would have liked due to your physical health?	81.7			18.3		
5. During the past 4 weeks, have you had to stop doing some tasks at work or in your daily activities because of your physical health?	85.3			14.7		
6. During the past 4 weeks, have you done less than you would have liked to do because of any emotional problems (such as feeling sad, depressed, or anxious)?	80.6			19.4		
7. During the past 4 weeks, have you not done your work or daily activities as carefully as usual because of any emotional problems (such as feeling sad, depressed, or anxious)?	82.7			17.3		
	Not at all	A little	Moderate	Quite a bit	A lot	
8. To what extent has pain made it difficult for you to do your usual work (including work outside the home and household chores)?	62.1	22.2	8.5	5.1	2.2	
	Never	Only sometimes	Sometimes	Often	Almost always	Always
9. How often have you felt calm and peaceful?	2.4	8.6	28.4	45.8	0.0	14.7
10. How often have you had a lot of energy?	3.4	12.3	35.2	37.8	0.0	11.2
11. How often have you felt discouraged and sad?	19.1	40.2	30.4	9.1	0.0	1.2
12. How often have physical health or emotional problems made it difficult for you to participate in social activities (such as visiting friends or family)?	43.3	7.8	45.7	0.0	2.6	0.6

Data are given in percentages (%). SF-12 score (physical dimension + mental dimension, 0–100 p): total: 64.1; male: 64; and female: 64.2. SF-12 score (physical dimension): total: 33.5; male: 33.3; and female: 33.6. SF-12 score (mental dimension): total: 30.6; male: 30.7; and female: 30.6. V. good: very good.

**Table 6:** Description of the most relevant characteristics of the participants based on the variables with the greatest importance for the computational metabotype algorithm.

	Westernized Millennial	Healthy	Active Mediterranean	Dysmetabolic/ Pre-Morbid	Metabolically Vulnerable/Pro-Morbid
**Frequency (%)**	28	25	17	18	12
**Age (years)**	39.8 ± 15.9	43.1 ± 14.6	47.2 ± 15.9	56.8 ± 15.7	59.1 ± 16.4
**Female (%)**	55.15	90.2	18.74	85.07	35.96
**Employment**					
Unemployment	6.04	2.98	1.98	5.07	8.36
Full-time student	17.43	9	6.84	2.29	1.74
Permanent illness/disability	0.89	0.22	0.55	2.09	3.94
Retired	5.66	6.46	14.77	32.64	30.13
Homemaker	2.48	4.35	1.54	10.75	6.47
Paid employment	67.49	77	74.31	47.16	49.37
**Smoking**					
Non-smoker	68.13	79.32	69.46	65.67	48.26
Ex-smoker	11.51	13.86	20.29	21.49	27.76
Current smoker	20.36	6.82	10.25	12.84	23.97
**Nap daily**	31.93	21.77	45.87	47.76	61.04
**Sleeping weekdays (7–8 h/d)**	62.02	66.26	60.31	49.95	47.79
**Sleeping weekends (7–8 h/d)**	53.88	62.92	63.73	52.44	49.21
**Disease**					
Obesity	1.34	0.44	9.7	15.12	39.27
T2D	2.04	1.16	7.28	19.2	28.08
HBP	6.74	5.15	20.18	39.9	50
Dyslipidemia	9.73	10.09	20.51	42.79	49.21
**BMI (kg/m^2^)**	23.6 ± 3.7	22.5 ± 6.4	26.4 ± 3.7	26.9 ± 3.7	30.7 ± 4.5
**Snacking (per day)**	53.18	30.55	29.55	40.4	68.61
**Meals (4 per day)**	30.28	35.41	36.49	31.94	26.81
**Use of salt habitually**	3.56	2.03	3.09	2.29	5.21
**MEDAS-17 p**	10.9 ± 2.1	6.3 ± 2.4	10.5 ± 2.1	6.8 ± 2.2	10.9 ± 2.1
**Characteristic food (%)**					
Whole milk (1–2/week)	3.63	1.67	2.21	1.59	5.52
Semi-skimmed milk (1–2/week)	4.07	7.91	6.84	9.35	5.36
Eggs (3–4/week)	15.78	14.66	24.04	12.94	19.72
Lean meats (3–4/week)	14.69	10.23	17.86	10.85	14.67
Fatty meats (1–2/week)	23.03	5.01	10.14	7.46	32.18
Whitefish (1–2/week)	8.72	22.35	25.91	26.37	11.67
Fatty fish (1–2/week)	5.98	14.44	17.42	11.04	7.26
Vegetables (1–2/day)	1.91	11.10	7.72	7.36	0.95
Fruits (3–4/day)	1.08	3.77	6.84	3.18	1.26
Nuts (+5/week)	13.17	32.95	34.18	24.98	10.09
Legumes (3–4/week)	2.10	4.79	5.51	3.18	3.31
Olive oil (+5/day)	2.42	2.76	4.63	3.38	2.68
Other fats (3–4/week)	16.16	5.44	4.96	4.58	18.45
Refined grains (1–2/week)	48.60	24.89	29.44	32.14	50.16
Whole grains(1–2/week)	15.90	47.31	41.90	35.22	9.46
Pastries (1–2/week)	37.47	7.91	7.94	11.34	45.74
Sugars (1–2/week)	61.32	22.71	24.81	26.87	62.30
Alcohol (1–2/week)	22.33	11.18	31.64	14.93	39.91
Water (+5/day)	36.07	56.10	57.66	41.79	25.55
**Health status perception (good or excellent)**	27.35	41.22	42.67	10.45	4.1
**Physical and psychological state**					
Limited by physical health?	10.43	3.99	5.51	43.98	46.69
Cut back on work or daily activities?	7	2.83	3.42	37.41	40.06
Limited by emotional issues?	17.75	8.56	4.63	38.61	37.7
Problems with work/daily activities?	16.22	7.98	3.53	33.93	33.6
**Moderate PA (d/week)**					
1	13.49	15.02	14.44	10.05	12.93
2	16.98	19.23	16.98	14.53	13.41
3	11.45	14.37	16.1	10.25	8.04
4	7.12	7.26	8.82	3.78	3.47
5	6.23	9.29	8.82	5.17	3.47
+5	7.44	10.3	16.54	7.96	4.73
No PA	37.28	24.53	18.3	48.26	53.94
**Time moderate PA**					
0–30 min/day	53.05	39.62	31.09	63.48	71.45
30–60 min/day	26.97	37.08	36.38	23.78	16.4
1–2 h/day	14.31	18.21	22.93	10.15	6.15
2–3 h/day	3.12	2.39	4.85	1.19	3.15
+3 h/d	2.54	2.69	4.74	1.39	2.84

BMI: Body Mass Index; HBP: high blood pressure; PA: physical activity; and T2D: diabetes mellitus.

## Data Availability

Data are contained within the article and Supplementary Materials.

## Supplementary Materials

The following supporting information can be downloaded at: https://www.mdpi.com/article/10.3390/medicina60040610/s1, Table S1. Listing of the 91 predefined groups derived from the questionnaires used in the PLENUFAR 7 project, resulting from the factor analysis. Table S2. Description of the beta coefficients (ß) to be assigned in the algorithm based on the type of variable and its response. Table S3. Probability of classification in the different clusters by participants of the PLENUFAR 7 project derived from the random forest analysis.
