# Supplementary material for: Nutritional and Lifestyle Features in a Mediterranean Cohort: An Epidemiological Instrument for Categorizing Metabotypes Based on a Computational Algorithm
Source: Medicina (Kaunas). 2024 Apr 8;60(4):610. doi: 10.3390/medicina60040610 (PMC11051796; doi:10.3390/medicina60040610)
Supplement: Supplementary file 1 [file medicina-60-00610-s001.zip › medicina-2906781-supplementary.pdf]

Supplementary Materials

Table S1. Listing of the 91 predefined groups derived from the questionnaires used in the PLENUFAR 7 project, resulting from the factor analysis.

1. Sex
2. Main Occupation
3. Weight (kg)
4. Height (cm)
5. Pant Size
6. Shirt Size
7. Perceived Health
8. What weight do you consider to be healthy for you (in kg)
9. How many kg do you think you should lose
10. How many kg should you gain
11. Race/Ethnicity
12. Educational Level
13. Smoking Habit
14. Whole Dairy
15. Semi/Skimmed Dairy
16. Eggs
17. Lean Meats
18. Fatty Meats
19. White Fish
20. Blue Fish
21. Vegetables
22. Fruits
23. Nuts
24. Legumes
25. Olive Oil
26. Other Fats
27. Refined Cereals
28. Whole Grain Cereals
29. Industrial Pastries
30. Sugars
31. Alcohol
32. Water
33. Wine (Do you drink wine, How much do you consume)
34. Living Situation
35. Obesity
36. Diabetes
37. High Blood Pressure
38. Dyslipidemia
39. Family History of Obesity
40. Family History of Diabetes
41. Family History of High Blood Pressure
42. Family History of Dyslipidemia
43. Have you felt sad, discouraged, low in spirits, or depressed
44. Should lose weight
45. Should gain weight
46. Take a nap
47. Do you usually snack or consume any food between meals
48. Do you use extra virgin olive oil
49. How many servings of vegetables
50. How many pieces of fruit (including juice)
51. How many servings of red meats
52. How many servings of butter
53. How many sugary drinks
54. How many servings of legumes
55. How many servings of fish or seafood
56. How often do you consume pastries
57. How often do you consume nuts
58. Do you preferably consume lean meat
59. How many times a week do you consume cooked meals
60. Do you add sugar to your drinks
61. How many servings of white bread
62. How many servings of cereals and grains
63. How many servings of bread, rice, and/or pasta
64. Nap Hours on weekdays
65. Nap Hours on weekends
66. Sleep Hours per night on weekdays
67. Sleep Hours per night on weekends
68. How many years have you smoked or been smoking
69. How many cigarettes do/did you consume per day

70.	Number of meals per day
71.	Do you use salt at the table when eating
72.	Glasses of water per day
73.	How would you rate your health compared to people your age?
74.	Moderate Efforts, such as moving a table, vacuuming, playing bowling
75.	Climbing several floors by stairs
76.	Did you do less than you would have liked due to your physical health?
77.	Did you have to stop doing some tasks at work or in your daily activities
78.	Did you do less than you would have liked due to some emotional problem (sadness
79.	Did you not do your work/daily activities as usual due to some problem
80.	To what extent has pain made your usual work difficult (including housework,
81.	How long have you usually felt calm and peaceful?
82.	How long have you usually had a lot of energy?
83.	How long have you usually felt discouraged and sad?
84.	How often have physical health or emotional problems made it difficult for you
85.	In a normal week, how many days do you do intense physical activities?
86.	How much total time do you usually spend on intense physical activity?
87.	In a normal week, how many days do you do moderate physical activities?
88.	How much total time do you usually spend on moderate physical activity?
89.	In a normal week, how many days do you walk for at least 10 consecutive minutes?
90.	How much total time do you usually spend walking on one of those days (h/day)?
91.	In a normal week, how much time do you spend sitting during a workday (h/day)?

Table S2. Description of the beta coefficients (ß) to be assigned in the algorithm based on the type of variable and its response.

Beta coefficient (ß)	Variable	Type of item
n1	Sex	• Male: 0<br>• Female: 0.0333
n2	Occupation	• Unemployment: 0<br>• Full-time student: –0.0403269<br>• Permanent illness disability: –0.1127479<br>• Retired: 0.024143<br>• Homemaker: 0.0383089<br>• Paid employment: –0.0590874
n3	Education	• Primary Education or less: 0<br>• Secondary Education or High School: –0.0852415<br>• Professional training: –0.3086324<br>• University Education or more: –0.2239328
n4	Smoking	• Non-smoker: 0<br>• Quitter smoker: 0.081127<br>• Smoker: 0.0378946
n5	Cigarettes per day	• 0: 0<br>• 0-10 cigarettes: 0<br>• 11-20 cigarettes: 0.0207365<br>• >20 cigarettes: 0.040844
n6	Cohabitation	• Alone: 0<br>• Married/cohabitant: –0.0473799<br>• With children: –0.0960838<br>• With elderly: –0.0490989<br>• Other: –0.1287298
n7	Hours of nap during the week	• 0: 0<br>• <30 min/d: 0.3374745<br>• 30-60 min/d: 0.295221<br>• >60min/d: 0.0473886
n8	Hours of weekend nap	• 0: 0<br>• 30 min/d: 0<br>• 30-60 min/d: –0.0688041<br>• >60 min/d: 0.0825323
n9	Weekday sleep times	• <5h: –0.1979225<br>• 5-6h: 0<br>• 7-8h: –0.0167996<br>• 9-10h: 0.0256039<br>• >10h: –1.052469
n10	Weekend sleep times	• <5h: 0.1696096<br>• 5-6h: 0<br>• 7-8h: 0.0069012<br>• 9-10h: –0.0683897<br>• >10h: –0.1035694
n11	Obesity	• No: 0<br>• Yes: 0.3792537
n12	DMII	• No: 0<br>• Yes: 0.2788483

n[13]	Hypertension	• No: 0<br>• Yes: 0.3480222
n[14]	Dyslipidemia	• No: 0<br>• Yes: 0.3563483
n[15]	Self-perception of losing weight	• No: 0<br>• Yes: 0.3929361
n[16]	Self-perception of gaining weight	• No: 0<br>• Yes: –0.3489201
n[17]	Snacking	• No: 0<br>• Yes: –0.0552395
n[18]	Number of meals per day	• 1-2: 0<br>• 3: 0.1234634<br>• 4: 0.2261114<br>• 5: 0.3441373<br>• >6: 0.0945412
n[19]	Use table salt	• Never: 0.0447343<br>• Seldom: 0.0413415<br>• Sometimes: 0.0134115<br>• Often: 0<br>• Usually: 0.0550272
n[20]	Self-perception of health	• Bad: 0<br>• Not great: –0.2696505<br>• Good: –0.6935279<br>• Very good: –0.7753575<br>• Great: –0.8175701
n[21]	Disheartened or sad	• Never: –0.2047133<br>• Only ever: –0.1942602<br>• Sometimes: 0<br>• Many times: 0.1442038<br>• Always: –0.1599464

Table S3. Probability of classification in the different clusters by participants of the PLENUFAR 7 project derived from the random forest analysis.

	Probability of classification				
Clusters	Westernized Millennial	Healthy	Active Mediterranean	Dysmetabolic or pre-morbid	Metabolically vulnerable
Westernized Millennial	81.8%	7.5%	4.2%	2.8%	3.7%
Healthy	7.8%	82.2%	4.6%	0.6%	4.7%
Active Mediterranean	7.1%	7.3%	77.3%	3.0%	5.2%
Dysmetabolic or pre-morbid	7.3%	1.6%	4.6%	78.3%	8.1%
Metabolically vulnerable	5.6%	6.7%	4.8%	4.7%	78.2%
